# Successful use of transesophageal echocardiography for minimally invasive cardiac surgery after esophagectomy with gastric tube reconstruction via the retrosternal route: a case report

**DOI:** 10.1186/s40981-026-00855-7

**Published:** 2026-03-26

**Authors:** Kanon Yokoi, Sakurako Kitade, Shoko Maruyama, Takayuki Yoshida

**Affiliations:** 1 Clinical Training Center, Kansai Medical University, Hirakata, Osaka Japan; 2 Department of Anesthesiology, Kansai Medical University Medical Center, 10-15 Fumizono-Cho, Moriguchi, Osaka 570-8507 Japan

**Keywords:** Transesophageal echocardiography, Esophagectomy, Gastric tube reconstruction, Retrosternal route, Minimally invasive cardiac surgery

## Abstract

**Background:**

The use of transesophageal echocardiography (TEE) after esophagectomy remains controversial. Herein, we describe a case in which TEE use was safe and effective during cardiac surgery after esophagectomy with retrosternal gastric tube reconstruction.

**Case presentation:**

A 71-year-old man, who had undergone esophagectomy with retrosternal gastric tube reconstruction, was scheduled for removal of a left atrial myxoma via right anterior thoracotomy under cardiopulmonary bypass. A multiplane TEE probe was smoothly inserted into the gastric tube, providing echocardiographic images similar to those acquired by transthoracic echocardiography via the parasternal and subcostal windows. TEE monitoring helped confirm the positions of the devices placed in the great vessels, tumor location, absence of residual air or leakage, and normal heart function. No complications related to the TEE were observed.

**Conclusions:**

Intraoperative TEE monitoring is a feasible option even in patients who have undergone esophagectomy with retrosternal gastric tube reconstruction.

**Supplementary Information:**

The online version contains supplementary material available at 10.1186/s40981-026-00855-7.

## Background

Transesophageal echocardiography (TEE) is essential for monitoring and decision-making during cardiac surgery [1]. However, whether TEE can be used in patients who have undergone esophagectomy remains controversial because of concerns regarding the safety and availability of ultrasound images [2, 3]. Here, we describe a case in which TEE was safely and effectively used to monitor the removal of a left atrial myxoma under cardiopulmonary bypass (CPB) in a patient with prior esophagectomy and retrosternal gastric tube reconstruction.

## Case presentation

The patient provided written informed consent for the publication of this case report.

A 71-year-old man (height, 160 cm; weight, 52 kg) was scheduled for surgical removal of a left atrial myxoma found on follow-up computed tomography (CT) after esophagectomy. Preoperative transthoracic echocardiography (TTE) showed an iso-echoic mass measuring 14.5 × 15.8 mm, attached to the fossa ovalis in the left atrium. Eight months earlier, the patient underwent robot-assisted esophagectomy for esophageal cancer with cervical anastomosis for retrosternal gastric tube reconstruction. Considering the possibility of tissue adhesion in the anterior mediastinum, the cardiovascular surgeons decided to perform surgery through a right anterior intercostal thoracotomy in the hemi-left lateral decubitus-position, thereby avoiding a midline sternotomy.

Preoperative CT revealed no strictures at the cervical anastomosis site. A gastrointestinal surgeon advised that an 8-month interval after esophagectomy in this case would be sufficient to insert a TEE probe if there was no resistance to its advancement. We explained the potential risks and benefits of intraoperative TEE to the patient and obtained written consent for its implementation.

**Fig. 1:**
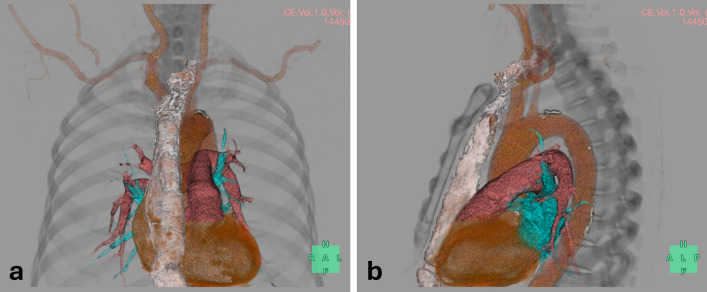
Three-dimensional reconstruction image from preoperative enhanced computed tomography. The spatial relationship between the retrosternal gastric tube (light pink) and heart is demonstrated. **a** Anterior view, **b** Left lateral view

**Fig. 2:**
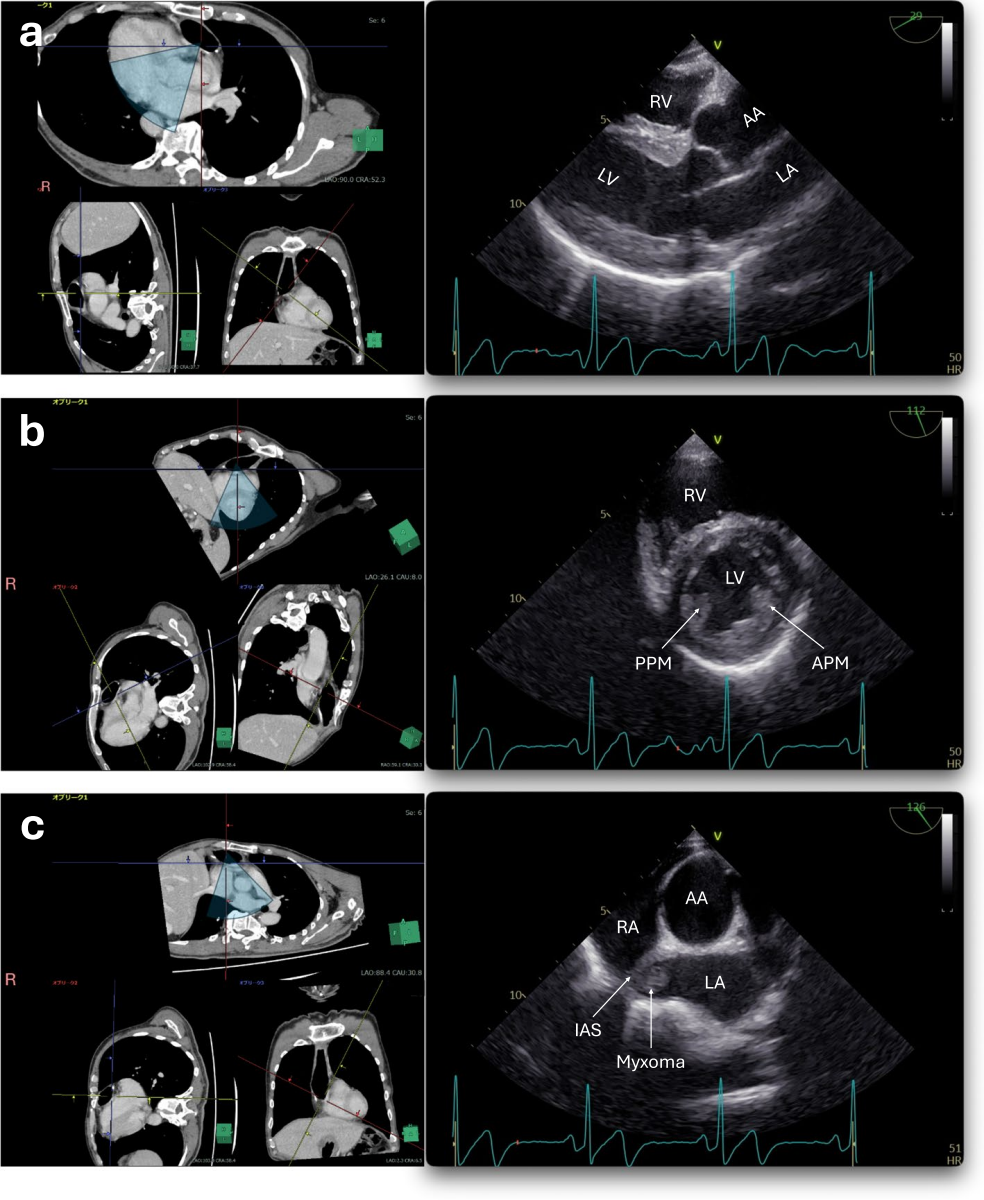
Transesophageal echocardiographic images, similar to left parasternal views, and corresponding sections on computed tomography. The right pane of each subfigure shows transesophageal echocardiographic images, while left pane shows corresponding sections on preoperative computed tomography (CT). **a** Long-axis view of the left ventricle, **b** Short-axis view of the left ventricle, **c** Short-axis view of the ascending aorta with a tumor seen in the left atrium. The inclinations of the yellow lines in the bottom-left and bottom-right CT images indicate the probe rotation and transducer array angles, respectively, when obtaining ultrasound images corresponding to the top CT images. The blue-shaded fan shapes in the top CT images replicate the ultrasound scanning area. AA, ascending aorta; APM, anterolateral papillary muscle; IAS, interatrial septum; LA, left atrium; LV, left ventricle; PPM, posteromedial papillary muscle; RA, right atrium; RV, right ventricle

After the induction of general anesthesia, the patient’s trachea was intubated, and we attempted to insert a multiplane TEE probe (6VT-D; GE HealthCare Japan, Tokyo, Japan). Preoperative CT indicated that the esophagus was located to the left and posterior to the trachea, immediately distal to the pharyngoesophageal junction, and then traveled anteroinferiorly and obliquely toward the retrosternal gastric tube situated in the midline (Fig. 1). The anastomosis between the residual esophagus and gastric tube was located in the midline of the anterior neck, deep to the manubrium. We initially inserted the slightly anteflexed tip of the TEE probe into the left side of the pharyngoesophageal junction using a video laryngoscope and advanced it distally to the right. Once the probe encountered mild resistance to its advancement around the midline, it was rotated 180° counterclockwise while releasing the anteflexion of the tip, after which it proceeded without further resistance. With the transducer lens oriented dorsally toward the heart, an echocardiographic image, similar to the parasternal long-axis view of the left ventricle in the TTE, appeared at a multiplane angle of approximately 30° (Fig. 2a). After advancing the TEE probe slightly further, an ultrasound image equivalent to the parasternal short-axis view of the left ventricle was observed at a multilane angle of approximately 115° (Fig. 2b). The “parasternal short-axis view” at the ascending aorta level visualized the intra-left atrium tumor attached to the interatrial septum (Fig. 2c).

**Fig. 3:**
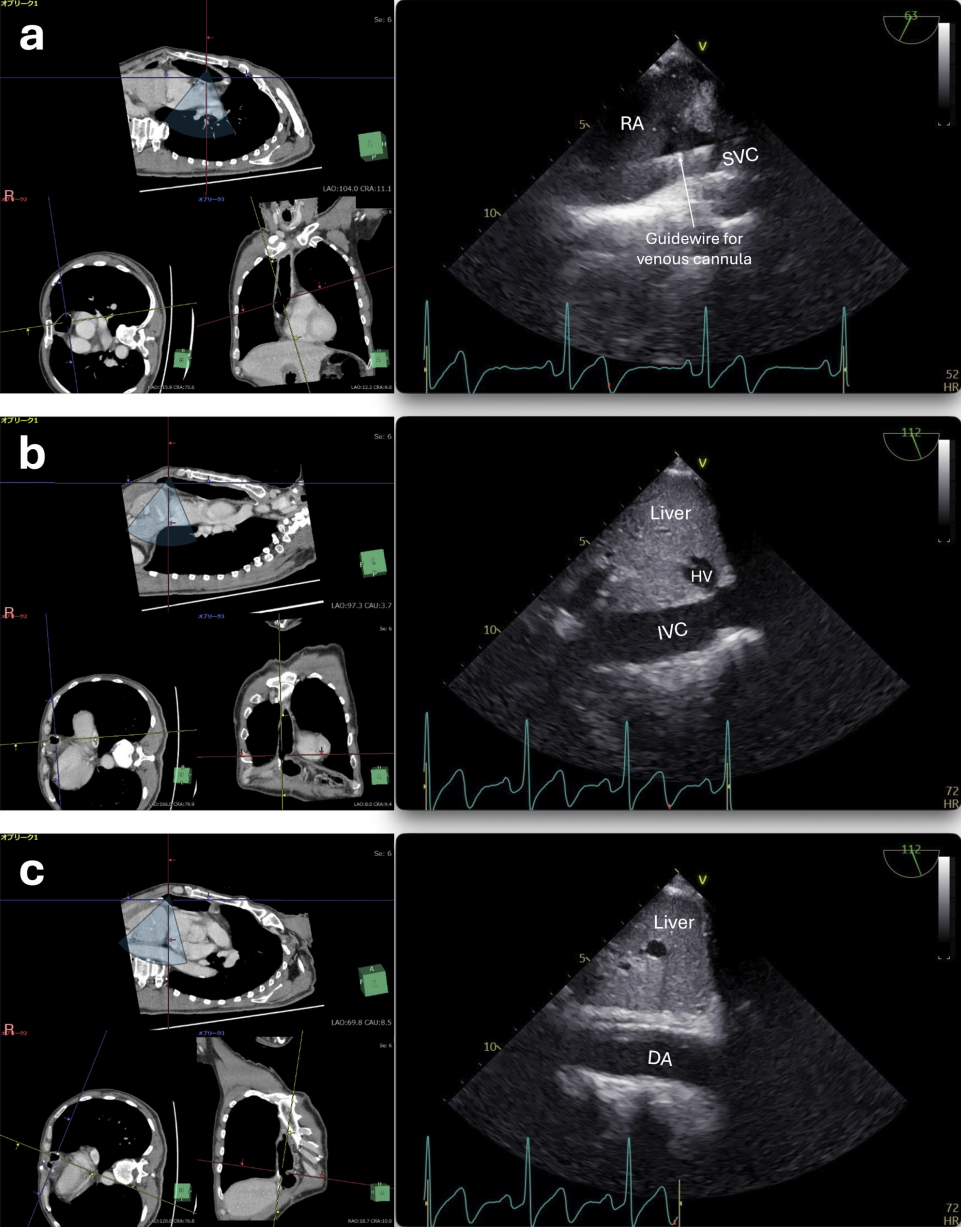
Transesophageal echocardiographic images, similar to right parasternal and subcostal views, and corresponding sections on computed tomography. The right pane of each subfigure shows transesophageal echocardiographic images, while left pane shows corresponding sections on preoperative computed tomography (CT). **a** Bicaval view with a guidewire in the superior vena cava, **b** Subcostal view of the inferior vena cava, **c** Subcostal view of the descending aorta. The inclinations of the yellow lines in the bottom-left and bottom-right CT images indicate the probe rotation and transducer array angles, respectively, when obtaining ultrasound images corresponding to the top CT images. The blue-shaded fan shapes in the top CT images replicate the ultrasound scanning area. DA, descending aorta; HV, hepatic vein; IVC, inferior vena cava; RA, right atrium; SVC, superior vena cava

The patient was positioned in the hemi-left lateral decubitus position, and surgery was initiated through right anterior thoracotomy in the fourth intercostal space. To establish CPB, the surgeons first attempted to insert a venous cannula from the right femoral vein toward the superior vena cava (SVC). We obtained a TEE equivalent to the right parasternal view of the TTE, showing the SVC, by rotating the probe counterclockwise at the level of the right atrium with a multiplane angle of 60°–70°. The guidewire inserted through the femoral vein was confirmed to have reached the SVC (Fig. 3a). Surgeons then tried to advance the venous cannula over the guidewire but encountered strong resistance in the iliac vein. The insertion site was then changed to the left femoral vein; however, the venous cannula still could not be advanced beyond the iliac vein. Subsequently, the surgeons placed venous cannulas in both the SVC and inferior vena cava (IVC) via the thoracotomy site. The IVC and descending aorta were visible on TEE, corresponding to the subcostal sagittal views of the TTE, obtained by advancing the probe beyond the diaphragm and rotating it counterclockwise and clockwise, respectively (Fig. 3b and c). The final positions of the two venous cannulas were confirmed using TEE (Supplementary Video 1), and an arterial cannula was placed in the right femoral artery.

After establishing a complete CPB and a bloodless surgical field, the surgeons opened the right atrium and accessed the left atrium through the interatrial septum. Following tumor removal, the defect in the interatrial septum was repaired using a patch of autologous pericardium. During CPB weaning, TEE monitoring confirmed the absence of residual air in the left heart system, the absence of a residual shunt around the repaired interatrial septum, and normal left ventricular volume and wall motion (Supplementary Video 1).

The TEE probe was removed smoothly at the end of the surgery (operative time, 192 min). The patient was transferred to the intensive care unit after tracheal extubation in the operating room. The patient tolerated soft and regular meals on postoperative days (PODs) 1 and 5, respectively, and was transferred to the ward on POD 5. The patient was discharged uneventfully from the hospital on POD 9. No signs of oropharyngeal or gastrointestinal injury due to the TEE probe, such as pharyngalgia, chest or abdominal pain, vomiting, hematemesis, and subcutaneous emphysema, were observed intraoperatively or postoperatively.

## Discussion

This is the first case report describing safe intraoperative TEE in a patient who had previously undergone retrosternal neoesophageal reconstruction. The TEE probe in the retrosternal gastric tube produced ultrasound images similar to those acquired using TTE.

In general, esophageal injury due to TEE probe manipulation has been reported to occur in 0.9–1.2% of cases [4, 5]. Severe complications from TEE are rare; for instance, gastroesophageal perforations occur at an incidence of 0.0022–0.05% [6]. In patients with prior esophageal surgery, the use of TEE raises two concerns: the safety and availability of ultrasound images. Smith et al. [2] retrospectively investigated 95 patients who underwent 145 TEE assessments with prior esophageal surgery and reported no serious complications, such as gastrointestinal perforation, sepsis, or death from TEE. However, 89% of the surgical procedures in the study were anti-reflux operations, and only seven participants underwent esophagectomy, specifically transhiatal or Ivor-Lewis procedures [3, 7]. Another retrospective review among 20,047 adult cardiac procedures over 15 years identified 20 patients with a history of esophagectomy prior to cardiac surgery [8]. Among the 20 patients, 12 had transhiatal esophagectomy, 2 had transthoracic esophagectomy (not specified as Ivor-Lewis or McKeown), and the procedure type was undocumented in 6 patients. A TEE probe placement was attempted in 16 patients, but was unsuccessful in 2 (12.5%); this rate of failure was higher than that in the general cardiac surgery population (0.18%) [9]. Furthermore, critical TEE views relevant to cardiac procedures were obtainable in only 71% of the 14 successful cases. No adverse symptoms attributed to the TEE probe placement were reported.

Our patient underwent a McKeown procedure, also known as a three-incision esophagectomy. This procedure involves a subtotal esophagectomy with anastomosis of the residual rostral esophagus to a reconstructed esophagus made of the stomach (i.e., a gastric tube) in the cervical region [7]. The gastric tube was pulled up to the cervical area via the retrosternal or posterior mediastinal route [7]. In patients with a posterior mediastinal gastric tube, the alignment with the heart is similar to that of the native esophagus. Hence, once the probe passes through the anastomosis, echocardiography can be performed, similar to conventional TEE [10]. However, high-quality “transgastric and deep gastric views” are often unavailable due to the absence of the stomach in its original position [8, 10].

In contrast, in patients who have undergone esophagectomy with retrosternal gastric tube reconstruction, the TEE probe must travel anteriorly from the oropharynx toward the retrosternal gastric tube in the cervical area through anastomosis. Endoscopists have reported that cervical anastomoses for retrosternal neoesophageal reconstruction could form at steep angles exceeding 45°, limiting endoscope maneuverability and requiring enhanced torque and tip deflection control [11]. Given that a TEE probe is inserted blindly, unlike endoscopic examinations, pre-insertion assessments of the oropharynx and neoesophageal anatomy on CT scans could help predict the ideal course of the probe, as in our case. A recent case series and narrative review of TEE in patients with prior esophagectomy also recommends preoperative CT to evaluate the anatomic positioning of the gastric conduit and potential regions of resistance encountered when advancing a TEE probe [6]. Moreover, real-time fluoroscopy may further facilitate precise probe guidance by correlating CT-derived anatomical information with probe positioning.

No consensus exists regarding the safety duration between esophagectomy and the first postoperative TEE. Endoscopists almost universally consider endoscopy performed 3 weeks or more after esophagectomy to be safe [12]. The anastomosis and reconstructed esophagus are vulnerable in the early postoperative period. An observational study of 80 esophageal reconstructions found that most anastomotic insufficiencies, including anastomotic leakage and conduit necrosis, occurred 2–7 days after esophagectomy [13]. Collectively, the 8-month interval between the esophagectomy and cardiac surgery in our patient was considered a reasonably safe period for TEE. Preoperative esophagogastroduodenoscopy is obligatory in patients at a higher risk of iatrogenic perforation, such as those with continued conduit-related complications or symptoms suggestive of anastomotic stricture [6]. In our case, this was not required, as none of these clinical signs were present preoperatively.

In this case, the TEE transducer with its lens oriented dorsally within the retrosternal gastric tube provided real-time echocardiographic images equivalent to those acquired using TTE. Notably, by rotating the probe clockwise and counterclockwise, both the left- and right-sided parasternal TTE images were obtained. Further probe advancement beyond the diaphragm level enabled visualization of the IVC and descending aorta through the liver, which appeared similar to the subcostal sagittal views of TTE. Another factor contributing to good ultrasound imaging via the retrosternal gastric tube was the surgical approach via a right anterior intercostal incision, which maintained the alignment between the gastric tube and cardiovascular system. Cardiac surgery with midline sternotomy may require pulling the gastric tube laterally to achieve a better surgical field, perhaps deteriorating ultrasound permeability from the probe through the gastric tube wall and pericardium toward the heart and great vessels.

An alternative method for intraoperative cardiac assessment is direct epicardial echocardiography using a sector probe on the surface of the heart, as described in a case report of mitral valve repair after esophagectomy with retrosternal reconstruction [14]. We also considered this technique as a backup plan, should TEE prove unsuccessful. However, a potential limitation of direct echocardiography is our unfamiliarity with the images obtained. In contrast, images attained via the TEE probe in the retrosternal gastric tube appear similar to TTE images and are easier to interpret.

In conclusion, intraoperative TEE through a retrosternal gastric tube after esophagectomy can provide TTE-like ultrasound images and may be safely performed during cardiac surgery under CPB via right anterior thoracotomy. Intraoperative TEE monitoring should be considered even in patients who have undergone esophagectomy with retrosternal gastric tube reconstruction when the benefits outweigh the risks.

## Supplementary Information

Supplementary Material 1. Video clips of intraoperative transesophageal cardiography. The first two clips confirm the placement of venous cannulas in the superior vena cava and inferior vena cava, respectively, before the initiation of cardiopulmonary bypass (CPB). The third to fifth clips taken during CPB weaning demonstrate the absence of residual air in the left heart system, the absence of a residual shunt around the repaired interatrial septum, and normal left ventricular volume and wall motion, respectively. AA, ascending aorta; CPB, cardiopulmonary bypass; IAS, interatrial septum; IVC, inferior vena cava; LA, left atrium; LV, left ventricle; RA, right atrium; RV, right ventricle; SVC, superior vena cava.

## Data Availability

Not applicable due to patient privacy concerns.

## Abbreviations

CPB: Cardiopulmonary bypass
CT: Computed tomography
IVC: Inferior vena cava
POD: Postoperative day
SVC: Superior vena cava
TEE: Transesophageal echocardiography
TTE: Transthoracic echocardiography
